# The IL-6/STAT3 pathway upregulates microRNA-125b expression in hepatitis C virus infection

**DOI:** 10.18632/oncotarget.24129

**Published:** 2018-01-10

**Authors:** Chia-Yen Dai, Yi-Shan Tsai, Wen-Wen Chou, Tawei Liu, Chung-Feng Huang, Shu-Chi Wang, Pei-Chien Tsai, Ming-Lun Yeh, Ming-Yen Hsieh, Ching-I Huang, Shang-Yin Vanson Liu, Jee-Fu Huang, Wan-Long Chuang, Ming-Lung Yu

**Affiliations:** ^1^ Hepatobiliary Division, Department of Internal Medicine, Kaohsiung Medical University Hospital, Kaohsiung, Taiwan; ^2^ Department of Occupational and Environmental Medicine, Kaohsiung Medical University Hospital, Kaohsiung, Taiwan; ^3^ Health Management Center, Kaohsiung Medical University Hospital, Kaohsiung, Taiwan; ^4^ Faculty of Internal Medicine, Kaohsiung Medical University, Kaohsiung, Taiwan; ^5^ Graduate Institute of Clinical Medicine, College of Medicine, Kaohsiung Medical University, Kaohsiung, Taiwan; ^6^ Center for Infectious Disease and Cancer Research, Kaohsiung Medical University, Kaohsiung, Taiwan; ^7^ Department of Marine Biotechnology and Resources, National Sun Yat-Sen University, Kaohsiung, Taiwan; ^8^ Institute of Biomedical Sciences, National Sun Yat-Sen University, Kaohsiung, Taiwan

**Keywords:** miR-125b, HCV, IL-6, STAT3, HCV

## Abstract

**Background/Aims:**

MicroRNA-125b (miR-125b) has been found to regulate inflammation and acts as an oncogene in many cancers. The mechanisms of miR-125b expression during hepatitis C virus (HCV) infection remain to be clarified. The present study aims to identify the factors that might regulate miR-125b expression in HCV infection.

**Results:**

High expression of miR-125b was found to correlate with HCV infection in replicon cells and in sera from HCV-infected patients, whereas the miR-125b inhibitor reduced HCV gene expression. The interleukin 6 (IL-6)/signal transducer and activator of transcription 3 (STAT3) pathway plays an inducible effect on miR-125b gene expression. STAT3 siRNA or inhibitor could reduce HCV replication.

**Materials and Methods:**

HCV replicon cells Con1 (type 1b) and Huh7/Ava5 (type 1b) were treated with 17-hydroxy-jolkinolide B (HJB) or STAT3 siRNA. Cell viability assay and Renilla Luciferase Assay were used. Fragments of the miR-125b-1 promoter were constructed for the luciferase reporter assay. PSMB8, PSMB9, miR-125b-1, and miR-125b-2 expression was determined using TaqMan^®^ Gene Expression Assays. Western blot analysis was performed to assess protein abundance.

**Conclusions:**

This study elucidates a novel pathway for miR-125b in the pathogenesis of chronic HCV infection and suggests it as a possible target for treating HCV infection.

## INTRODUCTION

Hepatitis C virus (HCV) is a major cause of chronic liver disease worldwide, chronically affecting more than 170 million people [1–3]. In the United States and most other developed nations, the prevalence of HCV infection is 1–2%. Our previous studies in Taiwan have revealed some HCV-hyperendemic villages in the southern area with anti-HCV prevalence greater than 20% [4, 5]. Although all oral direct antiviral agents (DAAs) reported recently have a high cure rate, the current standard treatment in Taiwan and in some Asian countries involves combination therapy with pegylated interferon-α (PEG-IFN) and ribavirin (RBV) for patients with chronic hepatitis C (CHC), with a relatively high sustained virologic response (SVR) rate of 75%–95% [6–8]. With the most recent advances in the use of effective DAAs, the treatment duration ranges from 8–24 weeks. However, PEG-IFN/RBV remains the standard treatment in countries where DAAs are not yet available.

MicroRNAs (miRNAs) are ∼22 nucleotide-long RNAs that silence gene expression post-transcriptionally and are suggested to play important roles in various processes, including intrinsic antiviral immunity [9] and HCV RNA replication [10]. MiR-125b is reported to function as a tumor suppressor in hepatocellular carcinoma (HCC) [11, 12] or as an oncogene in acute megakaryoblastic leukemia (AMKL) [13, 14] with multiple effects. A recent study showed that serum miR-125b levels were significantly higher in HBV or HCV-infected patients compared to those in healthy controls [15, 16], suggesting that miR-125b expression might be involved in viral hepatitis.

Interleukin 6 (IL-6) is an important cytokine in many inflammatory diseases. Serum IL-6 levels were reported to be significantly increased in several varieties of chronic liver diseases, including CHC [17]. The signal transducer and activator of transcription 3 (STAT3) is a transcription factor known to act downstream of the IL-6 pathway. STAT3 activation by tyrosine phosphorylation results in its nuclear translocation from the cytoplasm. Recent studies have shown that the HCV core activates STAT3 via an IL-6 autocrine pathway [18] and that JAK/STAT3 activation is enhanced in HCV replicon cells [19]. In addition, some studies have shown that STAT3 could regulate miRNA expression [20, 21]. Thus, a reciprocal regulatory relationship may exist among IL-6, STAT3, and miR-125b in HCV infection. On the other hand, a previous study has shown that the HCV core inhibits PSMB9 promoter activity [22]. PSMB9 is considered as an antigen-presenting gene that may be regulated by miR-125b during HCV infection. Since the underlying molecular mechanisms mediating IL-6-induced miR-125b expression during HCV infection are not fully understood, the present study aimed to explore whether miR-125b is upregulated by STAT3 activation and in turn regulates HCV replication. We also investigated the correlation between PSMB9 and miR-125b in HCV replicon cells to explore the biological roles of miR-125b in HCV replication through IL-6/STAT3 pathway activation and repression of the antigen-presenting gene, PSMB9.

## RESULTS

### Serum level of miR-125b is increased in HCV infection

**Figure 1:**
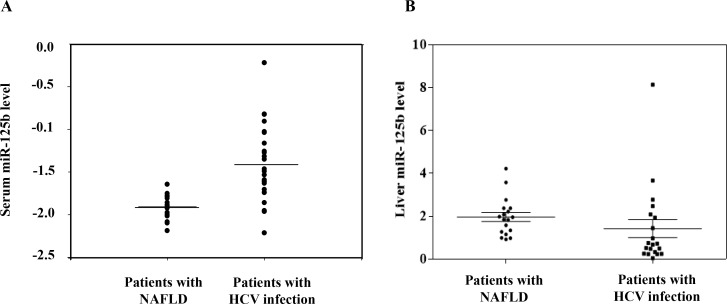
MiR-125b expression is higher in patients with HCV infection compared to that in patients with biopsy-proven nonalcoholic fatty liver disease The miR-125b expression levels in serum (**A**) and in liver tissues (**B**) were determined by real time PCR and were normalized to the corresponding geometric mean levels of snU6, miR-16, and miR-222 expressed as 2^-ΔCt^ where ΔCt = Ct (miR-125b)- Ct (geometric mean of control miRNAs). Each clinical sample was processed in duplicate.

To investigate whether miR-125b upregulation is associated with HCV infection, we examined the miR-125b levels in patients with chronic HCV infection. We found that miR-125b serum levels were significantly (*p* < 0.0001) increased among patients with chronic HCV genotype 1 infection compared to those with non-alcoholic fatty liver disease (NAFLD) (Figure 1; Table 1). But, hepatic miR-125b expression did not differ between patients with chronic HCV infection and NAFLD (Figure 1; Table 1).

**Table 1:** Clinical characteristics of individuals with nonalcoholic fatty liver disease (NAFLD) and with HCV infection in serum

	With NAFLD (*n =* 17)	With HCV infection (*n =* 25)	Univariate *p* value	Multivariate *p* value
Sex (M/F)	4/13	15/10	0.024	0.099
Age (years)	50.41 ± 14.97	51.84 ± 12.84	0.735	0.433
BMI (kg/m^2^)	22.33 ± 3.03	24.54 ± 3.10	0.036	0.657
Triglycerides (mg/dl)	87.47 ± 37.62	108.23 ± 43.27	0.135	0.434
Total cholesterol level (mg/dl)	177.18 ± 14.17	148.59 ± 34.65	0.002	0.056
Geometric mean of snU6, miR-16 and miR-222	26.88 ± 0.48	26.49 ± 0.95	0.131	-
2^-ΔCt(miR-125b-geometric mean)^	–1.91 ± 0.14	–1.41 ± 0.44	< 0.0001	0.047

Values expressed as mean ± SD (standard deviation); BMI, body mass index.

### MiR-125b increases HCV expression and miR-125b upregulation is mediated by STAT3

**Figure 2:**
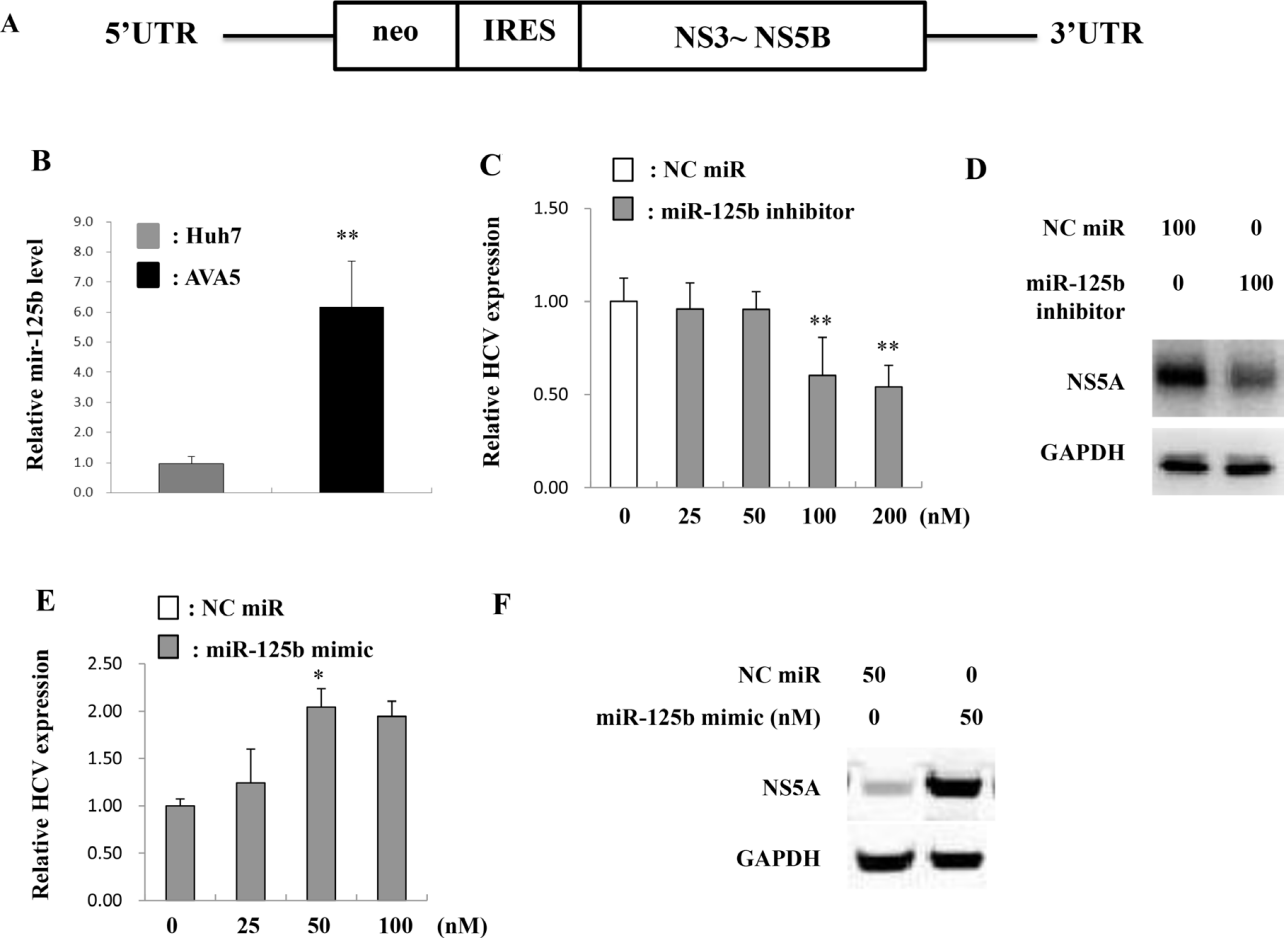
MiR-125b is upregulated in HCV infection (**A**) Genomic structure of HCV maintained in Ava5 cells. (**B**) Huh7 and Ava5 cells were incubated for 72 h and RNA was subsequently isolated at 72 h post-incubation. MiR-125b expression was determined by real time PCR and normalized to the corresponding level of snU6. The miR-125b inhibitor (**C**, **D**), miR-125b mimic (**E**, **F**), or negative control (NC) miRNA were respectively transfected into Ava5 cells. The HCV RNA and NS5A protein levels were determined at 72 h post-transfection by real time PCR and western blotting, respectively. GAPDH was used as the internal control. Data are expressed as means ± S.D. obtained from three experiments. ^*^P < 0.05 and ^**^P < 0.005.

**Figure 3:**
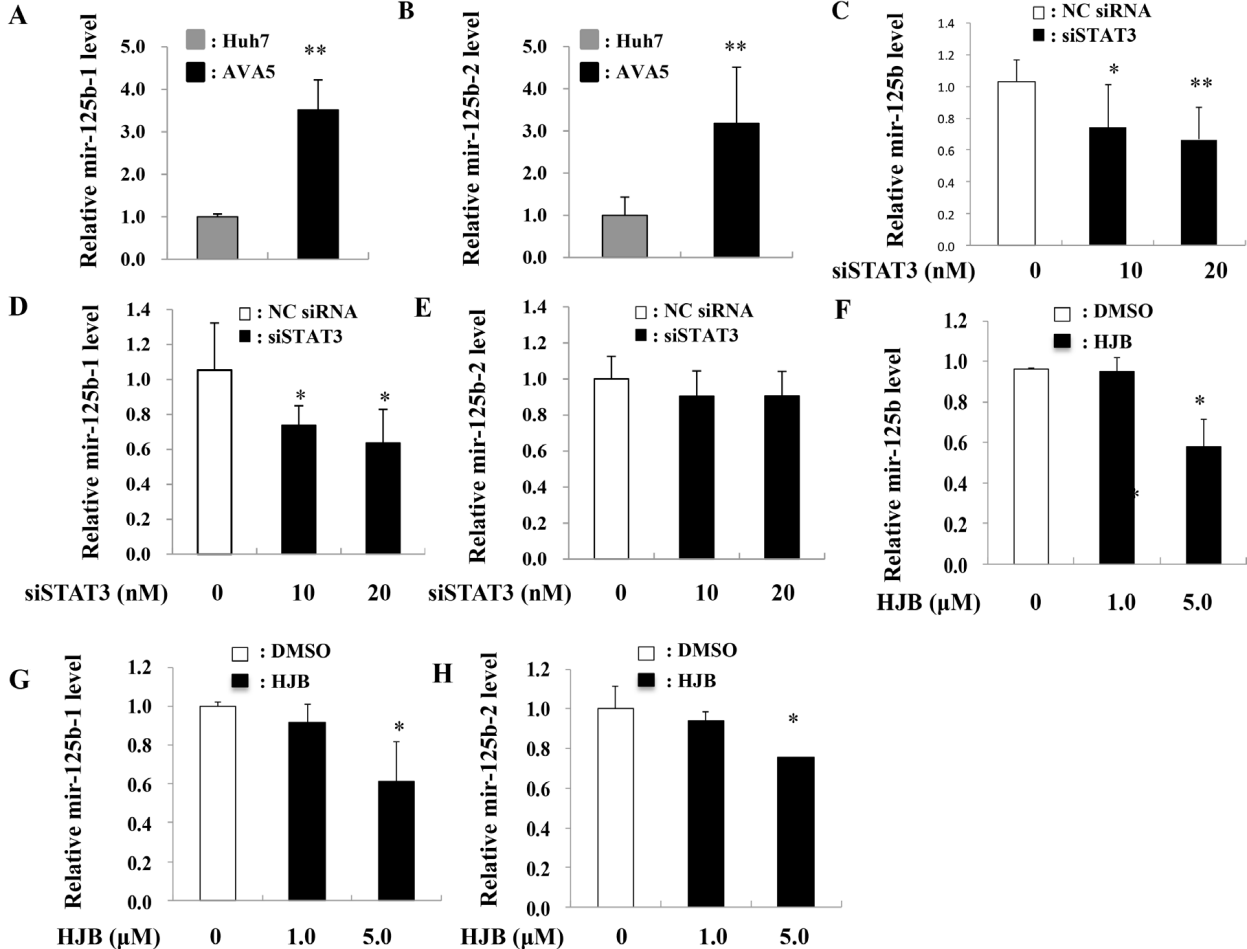
Inhibition of STAT3 reduced miR-125 levels (**A**, **B**) Expression levels of miR-125b-1 and miR-125b-2 in each sample were normalized to the corresponding levels of snU6. (**C**, **D**, **E**) siRNA-STAT3 (siSTAT3) or negative control (NC) siRNA was transfected into Ava5 cells, and RNA was isolated 48 h post-transfection. (**F**–**H**) Ava5 cells were treated with HJB, and RNA was isolated 24 h post-treatment. The expression of miR-125b, miR-125b-1, and miR-125b-2 was detected by real time PCR and normalized to the corresponding level of snU6. Data are expressed as means ± S.D. obtained from three experiments. ^*^P < 0.05 or ^**^P < 0.005 compared to the negative control group (NC) or HJB (0 μM) group, respectively.

To investigate the correlation between miR-125b and HCV infection, miR-125b expression in Ava5 replicon cells (genotype 1) was determined (Figure 2A). MiR-125b expression was significantly higher in replicon-bearing cells (Ava5) than in control cells (Huh7) (Figure 2B). To investigate whether miR-125b is associated with HCV replication, a miR-125b inhibitor or mimic was transfected into replicon cells and HCV gene expression was determined at 72 h after transfection. Real time-PCR and western blot analysis revealed that the miR-125b inhibitor decreased HCV expression at both RNA and protein levels (Figure 2C–2D). In contrast, the miR-125b mimic increased the HCV RNA and protein levels (Figure 2E–2F). Since miR-125b can be transcribed from two distinct loci on chromosomes 11 (hsa-miR-125b-1) and 21 (hsa-miR-125b-2), the expression levels of miR-125b-1 and miR-125b-2 were also determined. The expression of both miR-125b-1 and miR-125b-2 was increased in Ava5 replicon cells compared to that in Huh7 control cells (Figure 3A, 3B). In a previous study, we reported that miRNA expression can be regulated by transcription factors [23]. Using the TFSEARCH software and JASPAR database, a few putative STAT3 transcription factor binding sites on the miR-125b-1 or miR-125b-2 promoters were predicted. To determine whether STAT3 is associated with regulation of miR-125b, STAT3 siRNA transfection or treatment with the phospho-STAT3 inhibitor HJB was performed in Ava5 replicon cells, followed by determination of miR-125b expression. A previous study reported that HJB could strongly inhibit IL-6-induced STAT3 activation [24]. We first confirmed that STAT3 expression was inhibited by the STAT3 siRNA and that the phospho-STAT3 levels were decreased by HJB treatment (Supplementary Figure 1). We then found significantly decreased expression of miR-125b and miR-125b-1 by STAT3 siRNA transfection (Figure 3C–3D). However, miR-125b-2 levels were not altered after transfection with STAT3 siRNA (Figure 3E). Similarly, miR-125b and miR-125b-1 levels were decreased by treatment with the STAT3 inhibitor, HJB (Figure 3F–3G). However, miR-125b-2 expression was only slightly decreased by HJB treatment (Figure 3H). These results suggest that STAT3 increases miR-125b expression mainly through pre-miR-125b-1.

### Effect of IL-6/STAT3 activation on miR-125b expression

**Figure 4:**
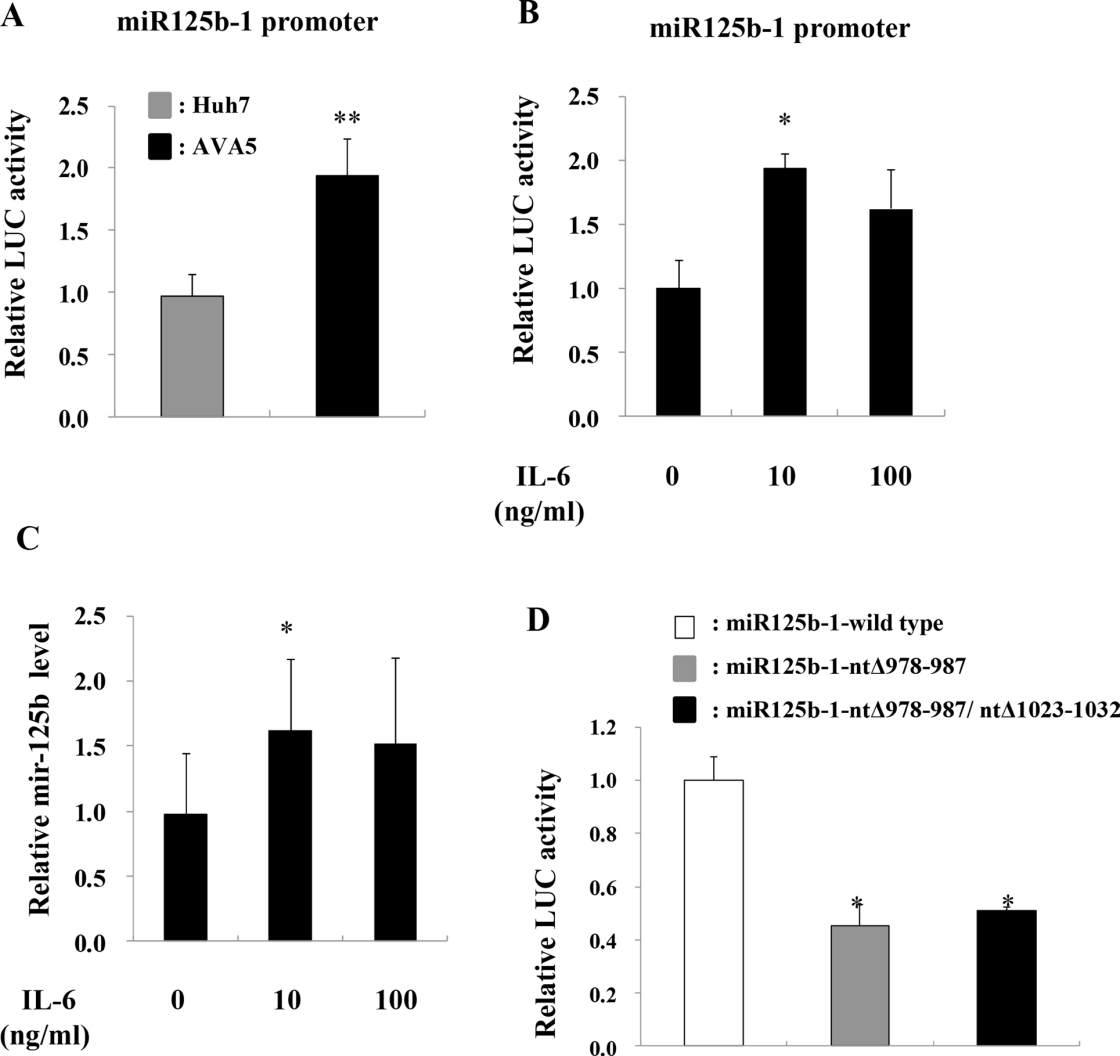
IL-6 induces miR-125b expression (**A**) Huh7 and Ava5 cells were transfected with the pGL3-Basic or pGL3-miR-125b-1 promoter plasmid for 24 h, and luciferase activity (Luc) was measured. (**B**) Huh7 cells were transfected with pGL3-miR-125b-1 promoter plasmid for 48 h, followed by rhIL-6 treatment for another 24 h, and luciferase activity (Luc) was then measured. (**C**) Huh7 cells were treated with rhIL-6 and RNA was isolated 24 h post-treatment. The expression of miR-125b was detected by real time PCR. (**D**) 293AD cells were transfected with wild-type pGL3-miR-125b-1 or mutant miR125b-1-ntΔ978-987 or miR125b-1-ntΔ978-987/ntΔ 1023-1032 plasmids for 48 h followed by treatment with rhIL-6 for 24 h, and luciferase activity (Luc) was then measured. The pEGFP plasmid was co-transfected into these cells, and GFP intensity was used as an internal control. Data are expressed as means ± S.D. obtained from three experiments. ^*^P < 0.05 and ^**^P < 0.005.

**Figure 5:**
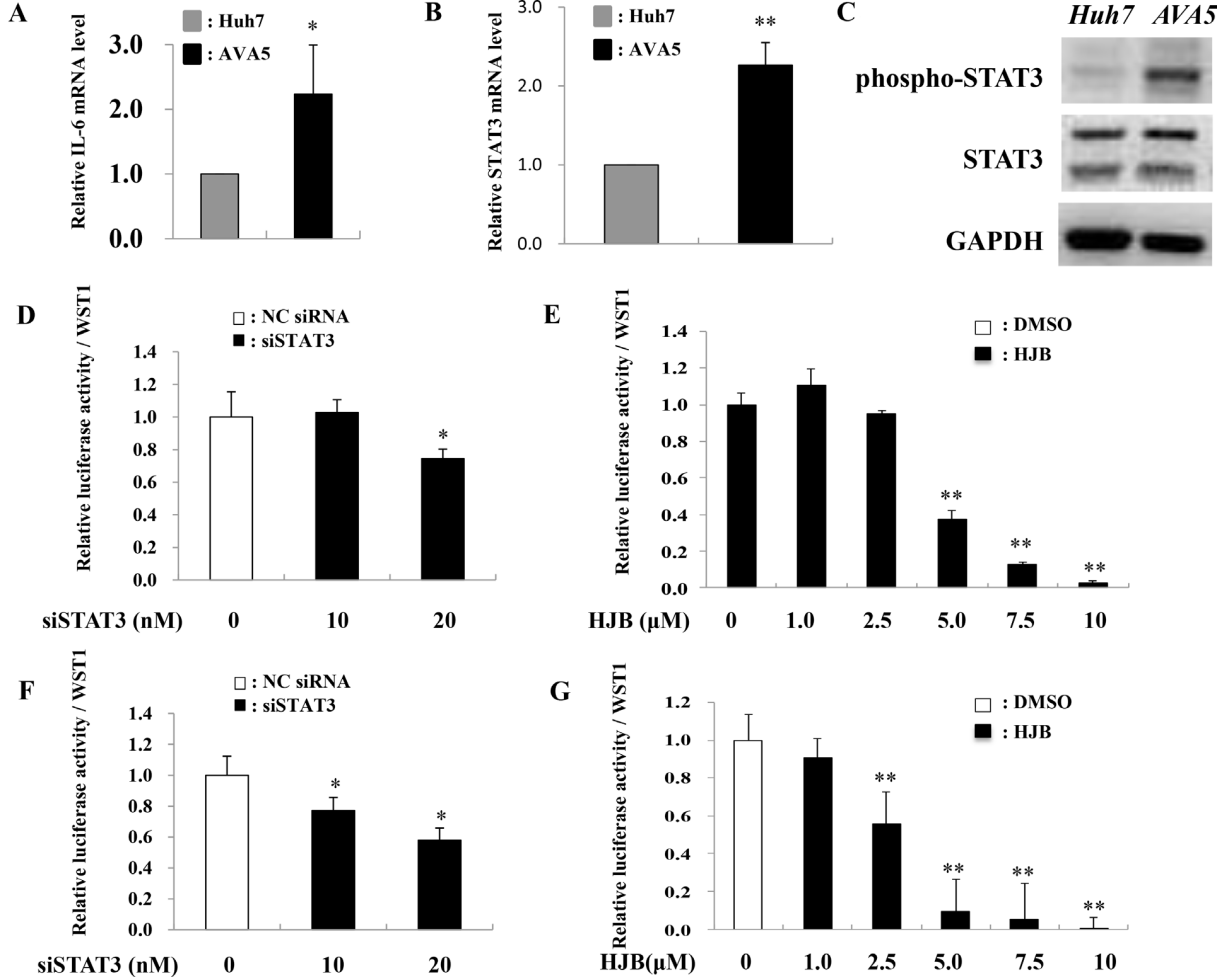
STAT3 knockdown reduces HCV translation (**A**–**C**) Huh7 and Ava5 cells were respectively incubated for 72 h, and subsequently subjected to RNA or protein isolation. IL-6 or STAT3 mRNA expression was determined by real time PCR. Phospho-STAT3 or STAT3 protein expression was measured by immunoblot analysis. (**D**, **E**) Con1 replicon cells and J6/JFH cells (**F**, **G**) were transfected with STAT3 siRNA or treated with HJB for 24 h. The Renilla luciferase reporter assay was performed 48 h post-transfection or 24 h post-treatment, respectively. Data are means ± S.D. obtained from three experiments; ^*^P < 0.05 and ^**^P < 0.005.

Since the STAT3-induced increase in miR-125b expression might occur via increased miR-125b promoter activity, a plasmid containing the miR-125b-1 promoter was constructed to investigate its promoter activity. Increased miR-125b-1 firefly luciferase promoter activity (LUC) was observed in Ava5 cells compared to that in Huh7 control cells (Figure 4A). Since IL-6 is upstream of STAT3 in the pathway, we determined whether miR-125b is upregulated by IL-6. The results showed that both miR-125b-1 promoter activity (Figure 4B) and miR-125b expression (Figure 4C) were increased by treatment with recombinant human IL-6 (rhIL-6) in Huh7 cells. Because the miR-125b-1 promoter sequence was predicted to contain two STAT3 transcription factor binding sites, the activated STAT3 may bind to the miR-125b-1 promoter and enhance its expression. We therefore deleted ten nucleotides from the two putative STAT3 binding sites (nt978-987 or nt978-987/nt1023-1032) on the miR-125b-1 promoter (Supplementary Figure 2). The activity of this promoter was significantly inhibited upon treatment with rhIL-6 in 293 AD cells (Figure 4D). To investigate the association between miR-125b upregulation and IL-6/STAT3, we examined the IL-6 or STAT3 expression in HCV replicon cells. The data revealed that IL-6 and STAT3 mRNA levels were significantly increased (Figure 5A, 5B) and phospho-STAT3 expression was significantly activated in Ava5 cells compared to that in Huh7 control cells (Figure 5C). To investigate the association between HCV replication and STAT3, Renilla luciferase activity was determined in Con1 (genotype 1b) replicon cells or in J6/JFH (genotype 2a) replicon cells (Supplementary Figure 3). We first checked the safety of the phospho-STAT3 inhibitor HJB, and observed that a dose of 5 µM did not cause cytotoxicity in Con1 cells (Supplementary Figure 4). Decreased luciferase activity was observed upon transfection of STAT3 siRNA (Figure 5D, 5E) or HJB treatment in Con1 replicon cells (Figure 5F, 5G).

### MiR-125b decreases PSMB9 expression in the HCV replicon model

**Figure 6:**
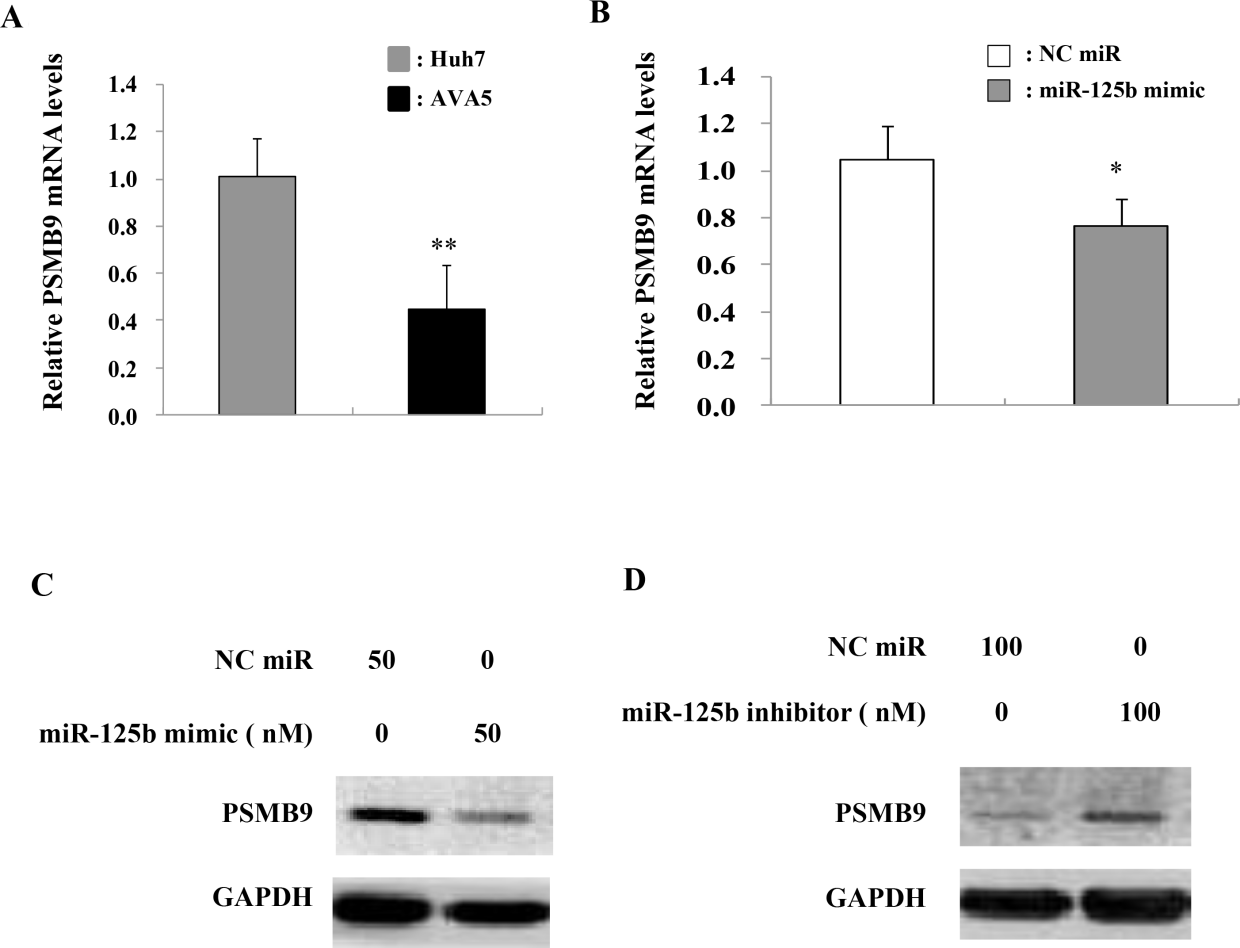
PSMB9 is targeted by miR-125b expression in HCV replicon cells (**A**) PSMB9 expression determined by real time PCR was significantly lower in HCV replicon cells compared to Huh7 cells. (**B**) The miR-125b mimic (50 nM final concentration), miR-125b inhibitor (100 nM final concentration), or negative control were transfected into Ava5 cells. PSMB9 mRNA and protein levels in Ava5 cells were then assessed by real time PCR and (**C**) immunoblot assay, respectively using GAPDH as an internal control. (**D**) MiR-125b inhibitor elevated PSMB9 expression.

**Figure 7:**
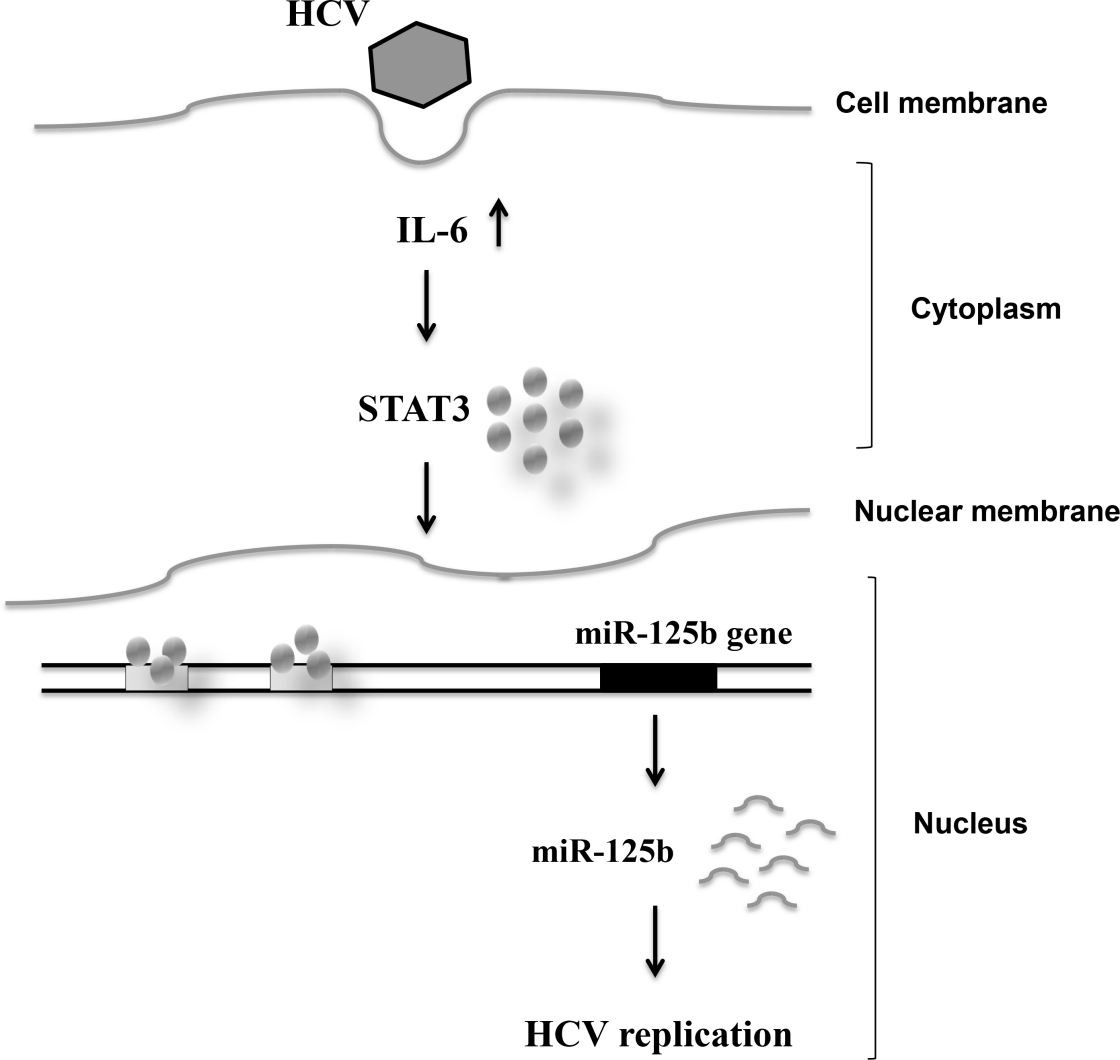
Schematic diagram showing the correlation among HCV infection, and IL-6/STAT3 and miR-125b expression

Since the antigen-presenting gene PSMB9 was one of the target genes of miR-125b, as determined by using TargetScan, MIRANDA, and miRWalk databases (Supplementary Figure 5), we compared PSMB9 mRNA levels in Ava5 cells and parental cells (Huh 7). The mRNA expression levels of PSMB9 were decreased in Ava5 cells compared to those in Huh7 control cells (Figure 6A). We then examined the effect of miR-125b on PSMB9 expression and found that transient transfection of the miR-125b mimic into Ava5 cells decreased the PSMB9 mRNA levels (Figure 6B). PSMB9 protein level was also decreased by the miR-125b mimic (Figure 6C) and was increased by the miR-125b inhibitor in HCV replicon cells (Figure 6D). However, the expression levels of PSMB9 were similar in the liver tissues from patients with HCV infection and with NAFLD (Supplementary Figure 6A). A significant inverse correlation between PSMB9 and miR-125b was only found in patients with NAFLD (Supplementary Figure 6B), but not in HCV infected patients (Supplementary Figure 6C). These results suggest that PSMB9 expression in HCV infected patients might not involve miR-125b alone. The schematic diagram of the correlation among HCV infection, IL-6/STAT3, and miR-125b expression is shown in Figure 7.

## DISCUSSION

MiRNAs like miR-122 are suggested to be associated with HCV infection and may have a role in regulation of HCV replication, thus rendering them possible alternative targets for HCV therapy [25]. We previously reported that the miRNA let-7g inhibited HCV replication resulting in reduced viral load and HCV gene and core protein levels, and it had an additive inhibitory effect with IFN/RBV on HCV replication [26]. Recently, we reported that miR-125b expression was increased in the peripheral blood mononuclear cells (PBMC) from patients with chronic HCV genotype-1 infection showing a non-sustained virologic response (non-SVR) [27]. It is unclear whether HCV infection induces overexpression of miR-125, resulting in reduced effectiveness of the anti-HCV treatment. We hypothesized that miR-125b might be upregulated in chronic HCV infection. In the present study, we found significantly increased expression of miR-125b in HCV replicon cells and in the sera of HCV-infected patients. The upregulation of miR-125b observed in our human serum samples from patients with HCV infection was similar to a previous finding of significantly increased miR-155 expression in HCV patients compared to that in normal controls [16]. However, the correlation between serum miRNA-125b levels and viral load in patients with CHC was not significant in the small sample population size (Supplementary Figure 7). Sampling error is a significant limitation of the standard liver biopsy, which represents the entire liver, resulting in absence of strong correlations between serum viral RNA levels, alanine aminotransferase (ALT) levels, and liver histopathology [28]. In fact, the level of functional mature miR-125b in three HCV replicon cells with stable expression of HCV non-structural proteins (Ava5, Con1, and J6) was still higher than that in uninfected cells ( Huh7 or Huh7.5) (Supplementary Figure 8). Therefore, miR-125b may be involved in the pathogenesis of chronic HCV infection.

We further investigated the mechanism underlying miR-125b upregulation in HCV infection. Since miR-125b overexpression was induced in HCV infection, we examined whether the miR-125b promoter could be regulated by transcription factors. We have previously reported that transcription factors could regulate miRNA promoters [23]. We have found a few putative STAT3 transcription factors predicted on the miR-125b promoter, using TFSEARCH software and the JASPAR database. STAT3, the downstream transcription factor of IL-6, is reported to be activated by HCV core or NS5A proteins [29–32]. McCartney *et al.* reported that HCV replication could induce STAT3 activation [33]. We demonstrated that STAT3 could bind the miR-125b promoter and regulate its expression in HCV replicon cells. In addition, HCV infection can induce inflammatory cytokine responses and enhance IL-6 production [34–36]. Elevated plasma and serum levels of IL-6 have been reported in HCV-infected individuals [17, 37]. We also found for the first time that IL-6 could increase the promoter activity and expression of miR-125b. On the other hand, exogenous miR-125b could induce STAT3 protein levels, STAT3 phosphorylation, and HCV non-structural protein expression, and could repress PSMB9 protein levels in an HCV replicon cell line (Supplementary Figure 9A, 9B), indicating a reciprocal regulation. Endogenous IL-6 RNA levels were not affected by exogenous miR-125b (Supplementary Figure 9C, 9D) implying that miR-125b expression was downstream of IL-6. Moreover, exogenous miR-125b also suppressed cell proliferation (Supplementary Figure 9E, 9F), suggesting that miR-125b affects host cells as well as HCV-infected cells (Supplementary Figure 10A, 10B). How exogenous miR-125b induces HCV non-structural protein expression is still unknown. Possibly, exogenous miR-125b could induce STAT3 expression and STAT3 phosphorylation, which may facilitate an increase in HCV replication [33, 38]. Several studies have shown that miR-125b promotes cell death or apoptosis by regulating the expression of Mcl-1, Bcl, IL-6R, and the spindle assembly checkpoint gene, MAD1 [39, 40]. Thus, miR-125b can affect HCV replication as well as host cell apoptosis.

Previous studies have indicated that HCV triggers formation of reactive oxygen species (ROS), leading to DNA damage and constitutive STAT3 activation [41, 42].

Manca et al. reported that miR-125b expression is increased by oxidative stress [43]. Therefore, STAT3-induced miR-125b expression might occur via triggering of the oxidative stress pathway by HCV. In addition, we found that both STAT3 siRNAs and inhibitors could decrease HCV replication in Con1 replicon cells. These results are similar to those of previous studies showing the role of STAT3 as a host factor in HCV replication [33, 38]. STAT3 inhibition markedly reduced HCV replication possibly through interaction with essential host cell factors or partly and indirectly through miR-125b expression. Considering the most recent report of a high response rate to combination therapy for 4 weeks with a miR-122 antagonist and DAAs [44], more efforts on the development of novel anti-HCV agents seem worthy. Our results on miR-125b and the IL-6/STAT3 pathway indicate a new possible target for further studies.

In summary, our study indicates an important role for miR-125b expression during HCV infection in replicon cells and clinical samples. The serum level of miR-125b was significantly elevated in HCV-infected patients. On the other hand, both the promoter activity and expression of miR-125b were increased in HCV replicon cells *in vitro*, whereas the miR-125b inhibitor reduced HCV expression levels. Our results also indicate that the IL-6/STAT3 pathway plays an inducible role in miR-125b expression. Taken together, we suggest that knockdown of STAT3 or miR-125b could be a promising strategy for anti-HCV therapy.

## MATERIALS AND METHODS

### Materials

All cell culture reagents were purchased from Gibco-BRL (Life Technologies, CA, USA). All chemical reagents were purchased from Sigma Chemical Co. (St. Louis, MO). The 17-hydroxy-jolkinolide B (HJB) reagent and chemiluminescence (ECL) solution were purchased from Millipore (Millipore, Billerica, MA). Primer sets were synthesized by Genomics Biotech (Taipei, Taiwan).

### Cell culture

The HCV replicon cells Con1 (type 1b), J6/JFH (genotype2a), and Ava5 (type 1b), and uninfected cells Huh7/Huh7.5/ Huh7.5.1 were obtained from Apath, LLC (St. Louis, Mo.) The sub-genomic HCV RNA was described previously [26]. These bicistronic replicon systems (Con1, J6/JFH, and Ava5) showed efficient delivery of adaptive mutations of the viral sub-genomic and full-length RNA with Renilla luciferase (RLuc-replicon) or neomycin resistance genes in G418 culture selection medium [45–47]. The 293 AD cell line was obtained from Cell Biolabs, Inc. (San Diego, CA, USA). Cells were cultured in Dulbecco’s Modified Eagle’s Medium supplemented with 10% heat-inactivated fetal bovine serum, 5% antibiotic-antimycotic solution, and 5% nonessential amino acid solution. Con1/J6 and Ava5 cells were maintained in complete media containing 0.5 and 1 mg/mL G418, respectively.

### Cell viability assay and Renilla luciferase assay

Cells were seeded into culture plates and treated with HJB or STAT3 siRNA. Viable cells were quantified using the WST-1 reagent (Clontech, CA, USA). Absorbance was measured on a microplate reader (BioRad, CA, USA). Con1 replicon cells expressing a Renilla luciferase reporter gene were seeded into culture plates and viable cells were quantified using the WST-1 reagent. Data from the WST-1 assay were derived from the same cells that were lysed to normalize Renilla luciferase activity (Promega Corporation, USA).

### MicroRNA and siRNA transfection

Mir-125b mimic, miR-125b inhibitor, and the miRNA NC (negative control) mimic and inhibitor were synthesized by RiBoBio (Guangzhou, China). The STAT3 and negative control siRNA were purchased from Sigma-Aldrich. MiRNA or siRNA were transfected into cells according to the manufacturer’s instructions using Oligofectamine™ as the transfection reagent (Life Technologies, CA, USA). Cells were harvested at 72 h after transfection and then used for experiments.

### Construction of miR-125b promoter and deletion reporter plasmids

The miR-125b-1 promoter fragments (2.5 kb) were constructed by Genewiz, Inc. (South Plainfield, NJ, USA) and the miR125b1-ntΔ978-987 or miR125b1- ntΔ978-987/Δ1023-1032 promoter plasmid was constructed by Protech Technology, Inc. (Taipei, Taiwan). For reporter assays, cells were transiently transfected with the wild-type or deletion reporter plasmids using Lipofectamine 2000™ (Life Technologies, CA, USA). The pEGFP plasmid was co-transfected to serve as an internal control. The reporter assay was performed 24 h post-transfection using the Luciferase Assay System (Promega Corporation, USA).

### RNA isolation and quantitative real-time PCR

Total RNA extraction was carried out according to the manufacturer’s instructions using TRIzol^®^ (Invitrogen). RNA quality was confirmed by A260/A280 measurement. cDNA was synthesized from 100 ng of total RNA using a High Capacity cDNA Reverse Transcription Kit (Applied Biosystems, CA, USA). PCR was performed in duplicate using the SYBR^®^ Green PCR Master Mix (Applied Biosystems) on the ABI Prism 7900 sequence detection system (Applied Biosystems) using the following primers: HCV sense primer, GGAAACCAAGCTGCCCATCA and HCV antisense primer, CCTCCACGGAT AGAAGTTTA. The expression levels of PSMB8, PSMB9, IL-6, and GAPDH were determined using TaqMan^®^ Gene Expression Assays. The expression ratios were calculated as the normalized CT difference between the control and the sample, and were adjusted for amplification efficiency relative to the expression level of the housekeeping gene GAPDH.

### MicroRNA assay

To detect miR-125b, cDNA was synthesized using a TaqMan MicroRNA Reverse Transcription Kit (Applied Biosystems). Relative miR-125b expression was determined on a Gene Amp 7900^®^ Sequence Detection System (Applied Biosystems). The expression levels of miR-125b in each sample were normalized to the corresponding level of snU6 or geometric mean of snU6, miR-16, and miR-122. The relative expression of miR-125b obtained from the serum and liver tissues was quantified according to the expression 2^-ΔCt^ using a logarithmic transformation. The expression levels of miR-125b-1 and miR-125b-2 were determined using TaqMan^®^ Gene Expression Assays. The expression ratios were calculated as the normalized CT difference between the control and sample and were adjusted for amplification efficiency relative to the expression level of the housekeeping gene, snU6.

### Western blot analysis

Western blot analysis was performed to assess protein abundance as described in our previous study (24). Anti-NS5A antibody was purchased from ViroStat, Inc. Anti-GAPDH antibodies were purchased from Millipore, Inc.

### Patients and clinical samples

We recruited 25 patients infected with HCV genotype 1 and 17 control patients with biopsy-proven nonalcoholic fatty liver disease (NAFLD) who were seronegative for anti-HCV antibodies, HCV RNA, and hepatitis B surface antigen (HBsAg). Protocols involving human subjects were approved by the ethics committee at the Kaohsiung Medical University Hospital and were conducted according to the guidelines of the International Conference on Harmonization for Good Clinical Practice. All participants provided informed consent.

### Statistical analysis

Frequency was compared between groups using the chi-square test with the Yates correction or Fisher’s exact test. Group means, presented as mean values ± standard deviation, were compared using analysis of variance and Student’s *t*-test or Mann–Whitney *U* test. A multivariate logistic regression model was used to determine the factors associated with outcomes. All *P* values are two-sided, and values less than 0.05 were considered statistically significant. All statistical calculations were performed using JMP software (version 9).

## SUPPLEMENTARY MATERIALS FIGURES



## Abbreviations

ALT: alanine aminotransferase
BMI: body mass index
CHC: chronic HCV infection
HCV: hepatitis C virus
HCC: Hepatocellular carcinoma
Huh7: Human hepatoma cell line
HJB: 17-hydroxy-jolkinolide B
Jak: Janus kinase
miRNA: Micro RNA
NAFLD: nonalcoholic fatty liver disease
SVR: sustained virologic response
STAT: Signal transducer and activator of transcription
