# Supplementary material for: The IL-6/STAT3 pathway upregulates microRNA-125b expression in hepatitis C virus infection
Source: Oncotarget. 2018 Jan 10;9(13):11291–302. doi: 10.18632/oncotarget.24129 (PMC5834265; doi:10.18632/oncotarget.24129)
Supplement: Supplementary file 1 [file oncotarget-09-11291-s001.pdf]

The IL-6/STAT3 pathway upregulates microRNA-125b expression in hepatitis C virus infection

SUPPLEMENTARY MATERIALS

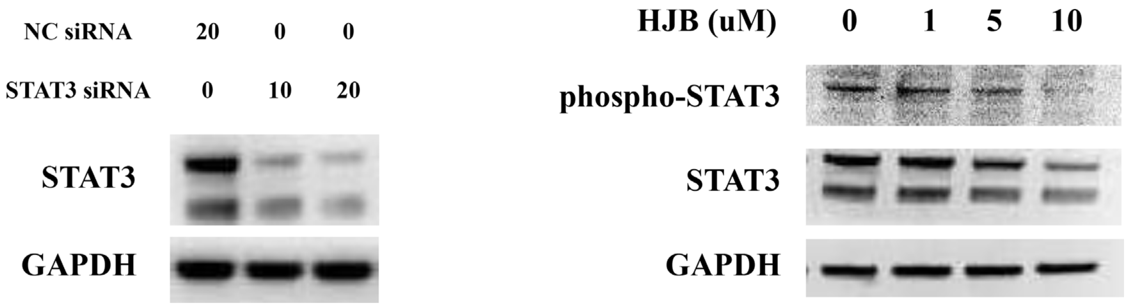

Supplementary Figure 1: STAT3 is specifically inhibited by STAT3 siRNA and the phosphorylation of STAT3 is inhibited by HJB.

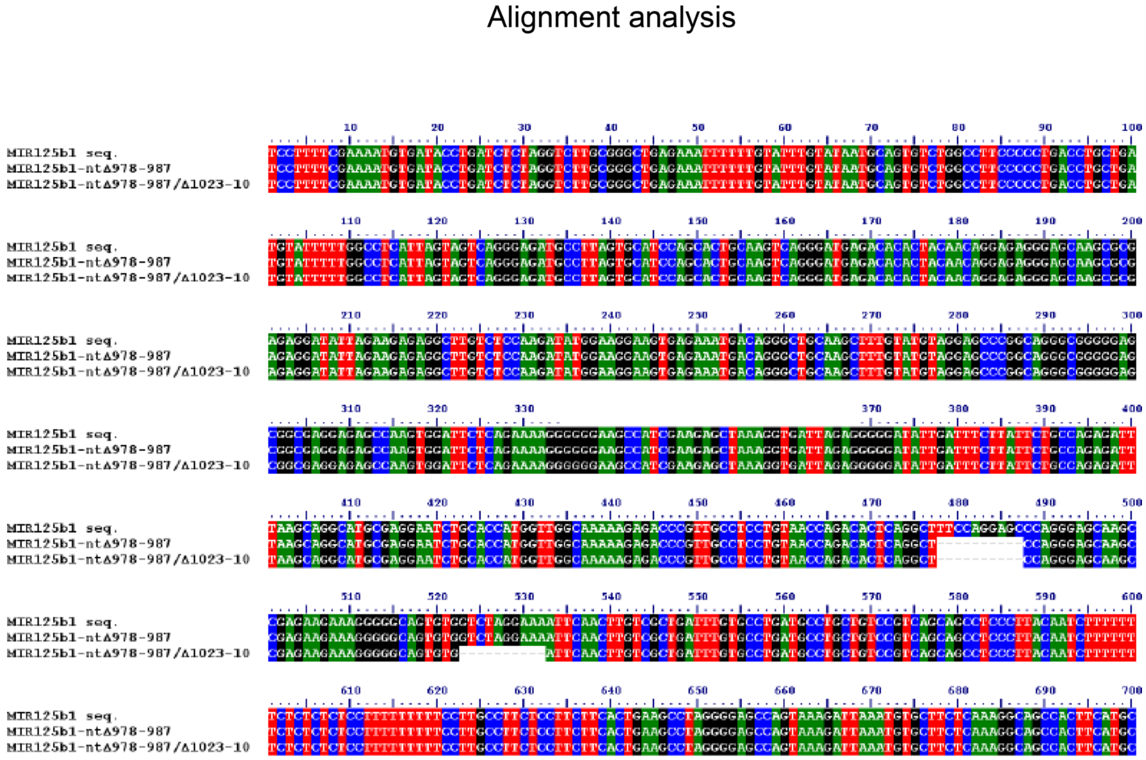

Supplementary Figure 2: The two putative STAT3 binding sites (nt978-987 or nt978-987/nt1023-1032) on the miR-125b-1 promoter.

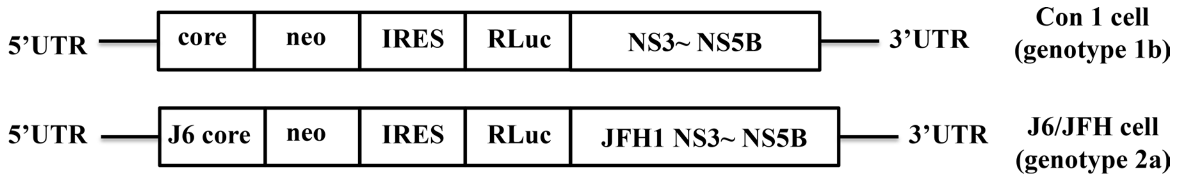

Supplementary Figure 3: Renilla luciferase activity was determined in Con1 (genotype 1b) or J6/JFH (genotype 2a) replicon cells.

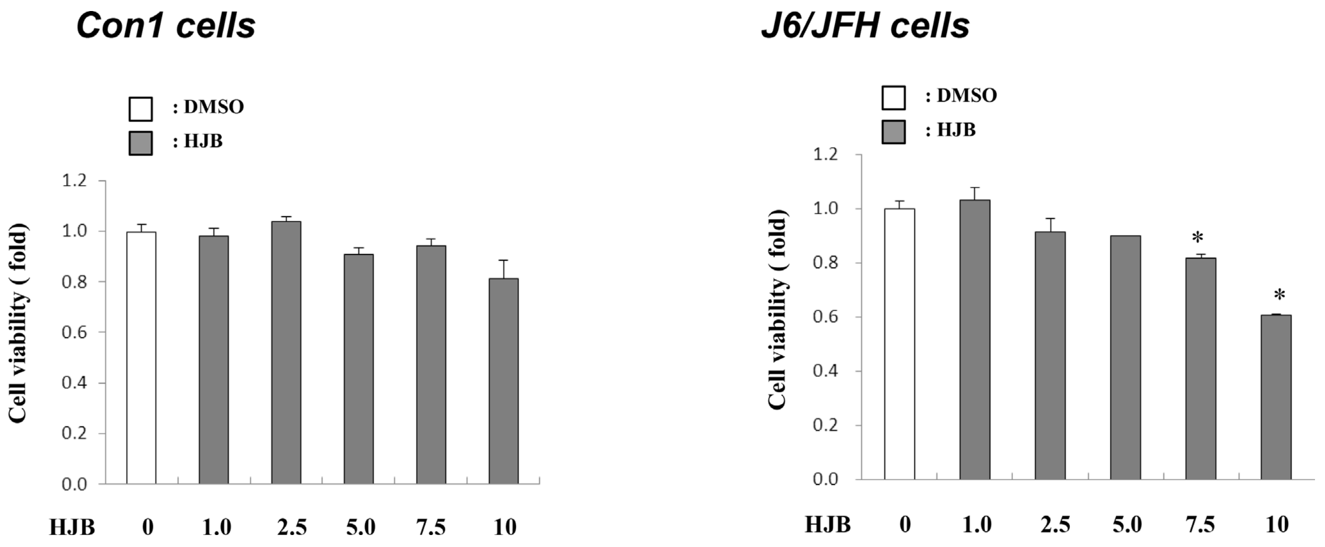

Supplementary Figure 4: Cytotoxicity of the phospho-STAT3 inhibitor, HJB, in Con1 or J6/JFH cells.

```
5'...AUUGGGAAAUGAGUGCUCAGGGA...(Position 81-88 of PSMB9 3' UTR)
                               |||||||
3'  AGUGUUCAAUCCCAGAGUCCCU  (hsa-miR-125b)
```

Supplementary Figure 5: PSMB9 was predicted as a potential target of miR-125b by TargetScan, MIRANDA, and miRWalk.

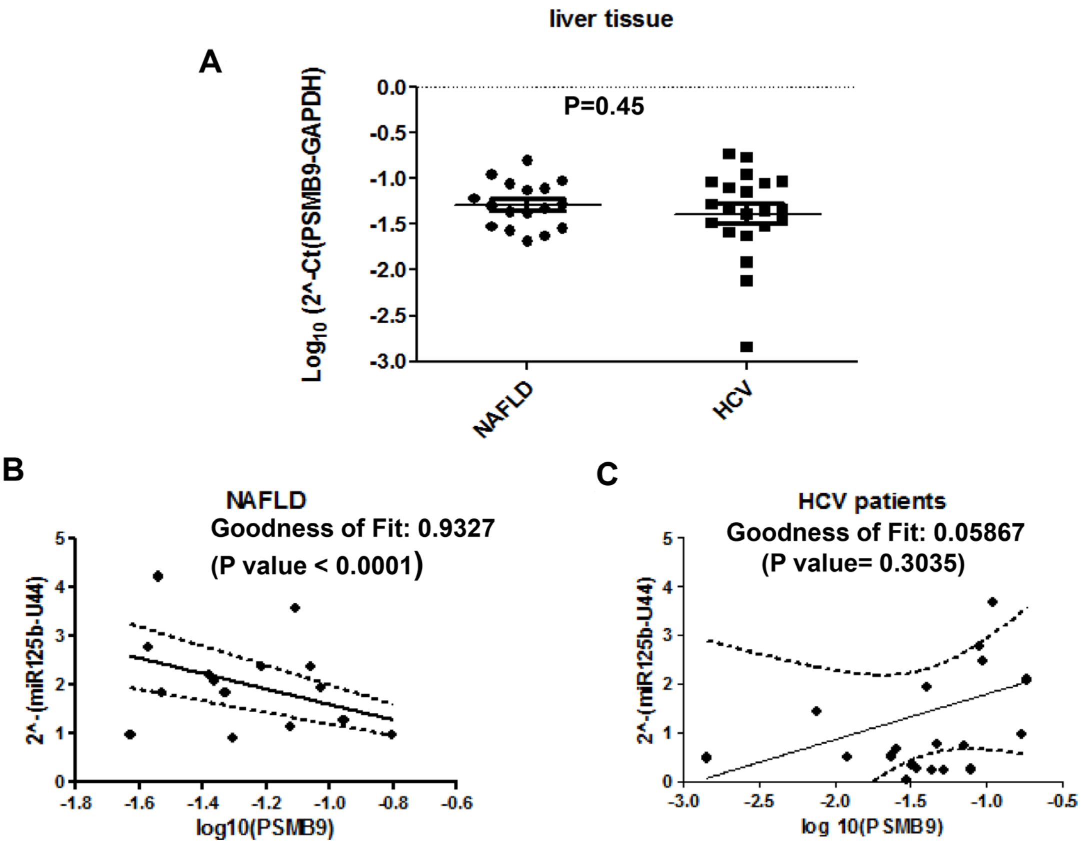

Supplementary Figure 6: PSMB9 expression levels in liver tissue were similar between patients with HCV infection and patients with NAFLD (**A**). A significant inverse correlation between PSMB9 and miR-125b was observed only in patients with NAFLD (**B**), but not in patients with HCV infection.

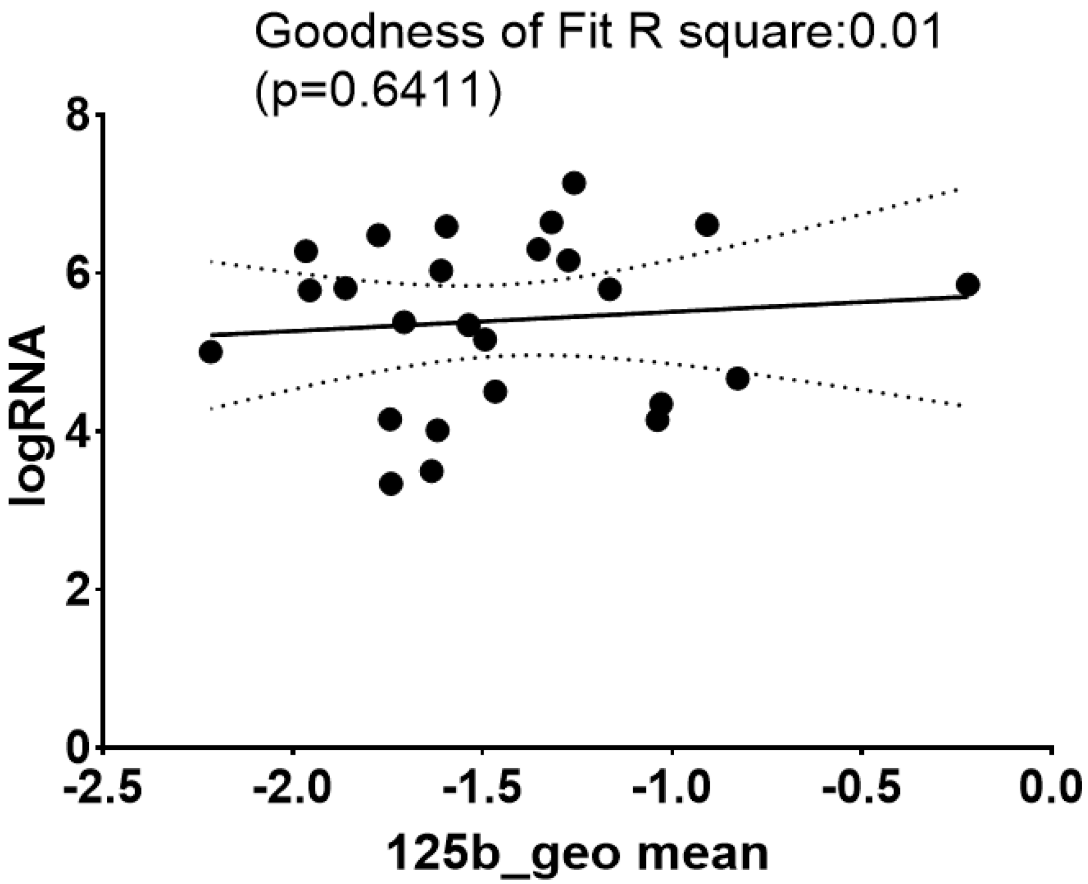

Supplementary Figure 7: Correlation between serum miRNA-125b levels and viral load in patients with CHC. Serum HCV viral load and miR-125b were analyzed using linear regression. The R-squared value was evaluated as a criterion of the goodness-of-fit test.

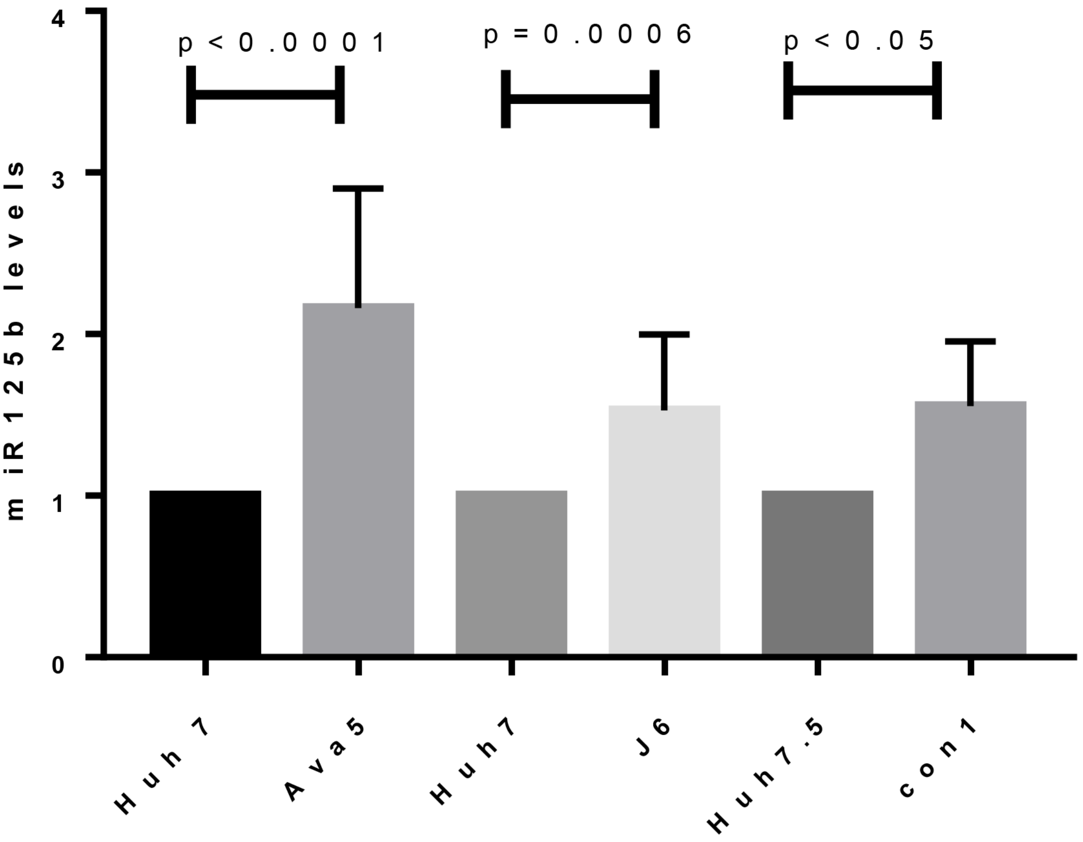

Supplementary Figure 8: Endogenous expression levels of miR-125b in hepatic cells. Total miRNAs were extracted and their expression levels were determined using TaqMan real-time PCR. U6 snRNA levels were used as internal controls. Data are expressed as means ± S.D. from three experiments. The relative expression was measured according to $2^{-\Delta\Delta Ct}$ relative quantitation method.

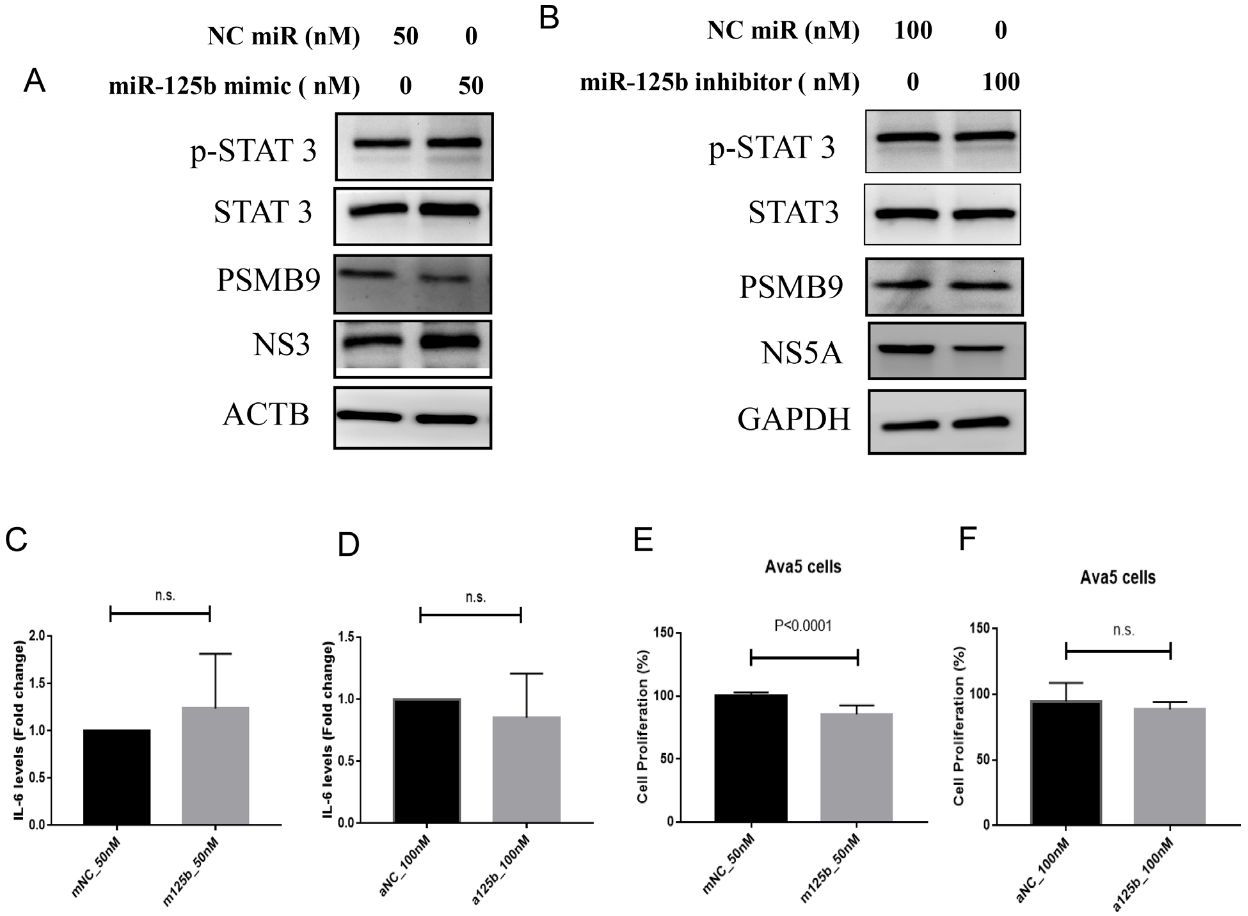

Supplementary Figure 9: MiR-125b downregulates PSMB9 and represses cell proliferation. PSMB9 protein levels in replicon cells (Ava5) were repressed by the miR-125b mimic (50 nM) and were increased by blocking the endogenous miR-125b using an inhibitor (100 nM) compared with the negative control (NC) in an immunoblot assay using beta-Actin as an internal control (**A**–**B**). The endogenous IL-6 level was measured by a Taqman gene expression assay (**C**–**D**). Cell proliferation was measured using the WST1 reagent. Data are means ± S.D. obtained from three experiments (**E**–**F**).

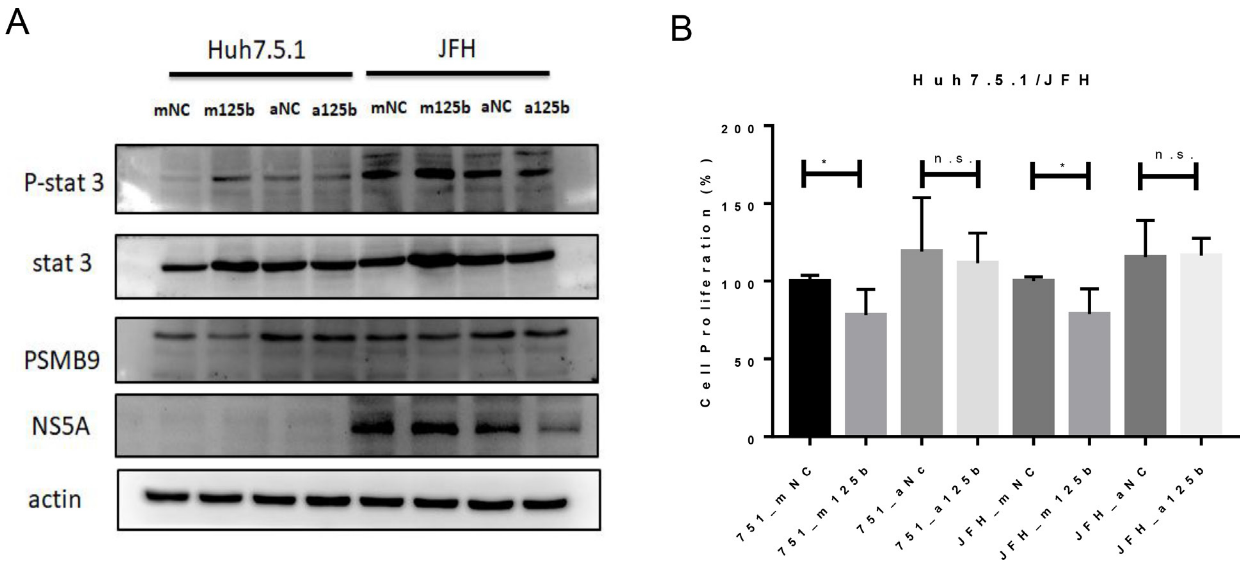

Supplementary Figure 10: MiR-125b induces STAT3 expression and phosphorylation in Huh7.5.1 and JFH cells. Total protein was isolated at 72 h post-infection and analyzed by western blot analysis using antibodies specific to p-STAT3 (Tyr705), STAT3, PSMB9, NS5A, and β-Actin, which served as a loading control (**A**). Cell proliferation was measured using the WST1 reagent. Data are means ± S.D. obtained from three experiments (**B**). Data are means ± S.D. obtained from three experiments. $^{*}P < 0.05$.
